# Perceived autonomy support from healthcare professionals and physical activity among breast cancer survivors: A propensity score analysis

**DOI:** 10.1371/journal.pone.0295751

**Published:** 2023-12-22

**Authors:** Audrey Plante, Lise Gauvin, Catherine M. Sabiston, Isabelle Doré

**Affiliations:** 1 Centre de Recherche du Centre Hospitalier de l’Université de Montréal (CRCHUM), Montréal, Canada; 2 École de Santé Publique de l’Université de Montréal, Montréal, Canada; 3 Faculty of Kinesiology and Physical Education, University of Toronto, Toronto, Canada; 4 École de kinésiologie et des sciences de l’activité physique, Faculté de Médecine, Université de Montréal, Montréal, Canada; Fred Hutchinson Cancer Research Center, UNITED STATES

## Abstract

The majority of women treated for breast cancer are physically inactive although physical activity (PA) could attenuate many adverse effects of cancer and treatment. Autonomy support from healthcare professionals may improve PA initiation, adherence and maintenance. This study aimed to determine, using a causal inference approach, whether or not perceived autonomy support (PAS) from healthcare professionals is associated with light, moderate, and vigorous intensity PA among women treated for breast cancer. Data were drawn from the longitudinal study “Life After Breast Cancer: Moving On” (n = 199). PAS was measured with the Health Care Climate Questionnaire and PA was assessed using GT3X triaxial accelerometers. Associations between PAS and PA were estimated with linear regressions and adjusted estimations were obtained using propensity score-based inverse probability of treatment weights (IPTW). Results reveal no association between PAS and PA of light ($\hat{\beta }$(95%CI) = -0.09 (-0.68, 0.49)), moderate ($\hat{\beta }$ (95%CI) = -0.03 (-0.17, 0.11)), or vigorous ($\hat{\beta }$(95%CI) = 0.00 (-0.03, 0.02)) intensity. Different forms of engagement and support by healthcare professionals should be explored to identify the best intervention targets to encourage women to adopt and maintain regular PA in the cancer continuum.

## Introduction

Improvements in cancer survival combined with the aging of the population will result in an increased number of women who have been treated for breast cancer in the coming years [1]. These women will experience many adverse effects of cancer and treatments (e.g., fatigue, pain, lymphedema, symptoms of depression and anxiety) that will reduce their quality of life [2].

In recent years, physical activity (PA) has been recognized as an effective and safe strategy to alleviate cancer- and treatment-related symptoms and to reduce the risk of developing other chronic diseases [2]. Despite these benefits, only 16–37% of women diagnosed with breast cancer meet PA recommendations [3–5]. While some breast cancer treatment regimens may induce more symptoms than others in patients, the literature suggests that it is the symptoms of cancer and its treatments that affect PA patterns in cancer survivors, rather than the treatment itself [6–8]. Symptoms of fatigue, pain and depression, in particular, are often cited as affecting PA and quality of life [2, 7]. These symptoms often lead to a lack of motivation for PA, which is one of the main barriers to PA reported by patients [9].

Healthcare interventions based on Self-Determination Theory (SDT) have shown promise in increasing motivation to adopt and maintain PA in patients [10]. According to SDT, motivation to initiate and maintain healthy lifestyles is acquired by the fulfillment of three basic psychological needs: competence, autonomy, and relatedness [11]. Although the fulfillment of all three psychological needs is desirable, the satisfaction of the need for autonomy (feeling of being the originator of the behaviour and that the action is voluntary) is essential in the development of autonomous motivation and the adoption of health-related behaviours [12]. Healthcare professionals such as physicians and nurses can support their patients’ autonomy and can even be trained to develop autonomy-supportive skills [13, 14].

Evidence shows that greater perceived autonomy support (PAS) from a physician or a nurse is associated with increased PA behaviour but few studies focus on women diagnosed with cancer and even fewer on the period just after diagnosis [15–18]. Thus, it is unknown whether autonomy support from healthcare professionals impacts PA participation soon after treatment is completed. The cross-sectional designs of most studies prevent causal inference in examining the association between autonomy support and PA. Longitudinal design and causal inference analytical strategies considering potential confounders are needed. Most of the studies use self-report measures to quantify PA; although inexpensive and widely used, these instruments are subject to recall bias which can lead to misclassification biases [19].

To overcome limitations from previous studies, we investigate the association between PAS from healthcare professionals and device-measured light, moderate, and vigorous intensity PA among women who had recently completed treatment for breast cancer using longitudinal data collected in the early months following treatment. A causal inference method was used, inverse probability of treatment weighting (IPTW), to control for confounders and to estimate the effect of exposure on the outcome with observational data. We hypothesize that a higher level of PAS will be associated with higher levels of light, moderate, and vigorous intensity PA.

## Methods

### Participants and data collection

**Fig 1:**
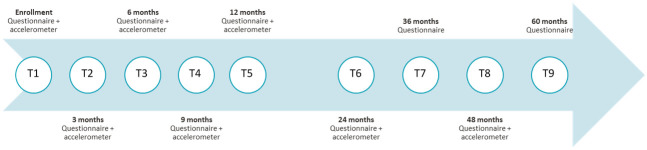
Illustration of data collection times for the "Life After Breast Cancer: Moving On" study (2010–2018).

Data were drawn from the *Life After Breast Cancer*: *Moving on* study longitudinal study [20]. Participants were recruited from 2009 through 2014 from hospitals and clinics in Montréal through targeted advertisements and referrals from oncologists. Participants had to meet the following inclusion criteria: 1) being at least 18 years of age; 2) having recently completed (0–20 weeks) primary treatment for stage I-III breast cancer diagnosis; 3) treated for a first cancer diagnosis (chemotherapy, radiotherapy, hormone therapy, surgery); 4) being able to provide written informed consent, read, and speak English or French; 5) reported no health problems that prevented from engaging in PA on the Physical Activity Readiness Questionnaire (i.e., high blood pressure, heart condition, balance or dizziness issues, bone, joint, or soft tissue problem) [21]. Participants were asked to provide data every three months during the first year (T1 to T5) and then once a year for four years (T6 to T9) for a total of nine data collections (Fig 1). The study methods are reported in greater detail elsewhere [20]. For the present study, data from the first 4 data collections were used to capture the early months post-treatment completion (T1 to T4–0, 3, 6 and 9 months after enrollment).

All participants provided written informed consent and procedures were approved by both the McGill University and Centre Hospitalier de l’Université de Montréal research ethics boards (project numbers A10-B14-08B and 20.131 respectively). The authors did not have access to any information that could identify individual participants during or after data collection.

### Measures

#### Participant characteristics

Participant characteristics at baseline included socio-demographic (age, ethnicity, education, annual family income, marital status) and clinical information (cancer stage, treatments received, end of treatment, BMI, smoking status). Data on physical and mental health prior to exposition (0–3 months after study inception) were also collected. Depressive symptoms were assessed using the Center for Epidemiological Studies Depression Scale, stress symptoms using the Perceived Stress Scale, cancer-related worries using the Assessment of Survivor Concerns Questionnaire, fatigue symptoms using the Brief Fatigue Inventory and self-reported pain and physical symptoms using a questionnaire based on the Primary Care Evaluation of Mental Disorders Screening [22–26]. All socio-demographic and clinical variables, as well as physical and mental health indicators variables mentioned above, were included in the propensity score estimation model because they are considered potential confounders or predictors of the outcome, as recommended for IPTW.

#### Light, moderate and vigorous intensity physical activity

PA was measured using the GT3X triaxial accelerometer (Actigraph, Pensacola, Florida, USA). Participants were required to wear the accelerometer on their hip for seven consecutive days, except at night and during water activities, and return it by mail to the research team. Accelerations (counts) were captured every 60-second interval. Using pre-established thresholds, counts were classified in time spent in light (100–1951 counts/min), moderate (1952–5724 counts/min), and vigorous (>5725 counts/min) intensity PA [27]. The outcomes of interest are the average proportion of accelerometer wear time spent in each PA intensity expressed as a percentage per day of light, moderate and vigorous intensity PA at T4 (9 months after enrollment) to account for variability in participants’ accelerometer wearing time. At T4, participants were, on average, 15 months post-treatment.

#### Perceived autonomy support from healthcare professionals

PAS was measured using the 6-item Health Care Climate Questionnaire (HCCQ—short) which has shown good construct validity and good internal consistency with patients with breast cancer [28]. Participants were asked to indicate on a 7-point Likert scale (1 = *not at all true*; 7 = *very true*) how supportive their healthcare providers were of their autonomy to engage in PA (e.g., I feel my healthcare providers understand how I see things with respect to my regular physical activity; My healthcare practitioners encourage me to ask questions about my physical activity). The questionnaire is available as supporting information. PAS from healthcare professionals was only measured at T3 (6 months after study inception and approximately one year after treatment completion) and was used as the continuous exposure of interest. To estimate propensity scores, which represent the probability of experiencing a high level of PAS, PAS scores had to be dichotomized. No information was found in the literature to help define a threshold beyond which autonomy support is considered “high”. Thus, the threshold was determined by the median of responses observed in the data (i.e., 3.5). Participants reporting a PAS of 3.5 or greater were considered exposed (i.e., high PAS) and participants with a PAS <3.5 were considered unexposed (i.e., low PAS).

### Analyses

Preliminary analyses included computing descriptive statistics. Missing data were handled with multiple imputation by chained equations (MICE) [29]. Variables in the imputation models were chosen using a correlation matrix (*r* > 0.1) and the classification and regression trees (CART) method was used to impute missing values [30]. There are no specific recommendations as to the exact number of imputations to be performed [31]. Previous recommendations suggested between 3 and 5 imputations, but these were deemed insufficient to avoid a decrease in power [32]. Graham et al. suggest taking into account the proportion of missing data and the tolerance for loss of power, and suggest a number of imputations based on this information [32]. Based on the proportion of missing data in our dataset (between 1.1% and 17.8%) and a power loss tolerance of less than 1%, the number of imputations was set at 20.

We used a propensity score analysis technique to overcome the methodological challenges of controlling for confounding factors and to balance the baseline characteristics of the participants, allowing causal inference [33]. Unlike other propensity score analysis techniques, such as matching, the IPTW method avoids excluding participants who cannot be matched from the analysis sample, and allows greater flexibility in the choice of main effect analysis [34]. The IPTW method involves creating a weighted sample with weights based on propensity scores. This method balances covariates (T1-T2) between exposed and unexposed individuals as if exposure had been randomly assigned [35]. The variables included in the propensity score estimation model were chosen according to the recommendations that suggest including 1) outcome measures that precede exposure, 2) variables that affect outcome without affecting exposure, and 3) variables that affect both outcome and exposure [36, 37]. The variables retained in the model are listed below (Table 1). We did not include the type of treatment received in the model because the literature shows that it is the symptoms of cancer and its treatments that affect physical activity more than the treatments themselves [6, 7]. Stabilized weights were based on propensity scores estimated using the Covariate Balancing Propensity Score method [38]. A weighted sample was generated in this manner in all 20 imputed data sets. To check whether the propensity score model was adequately defined, the balancing properties of the propensity score were checked in each of the 20 imputed data sets [33]. For each variable, the standardized mean difference (SMD) between the exposed and unexposed was evaluated. A variable with a SMD between -0.1 and 0.1 is considered balanced (i.e., exposed and unexposed are similar) [39]. Variable balance was achieved in each of the 20 imputed data sets.

**Table 1:** Variables included in the propensity score estimation model.

	Variables	Measured at T1	Measured at T2
Outcome measures that precede exposure	% light PA % moderate PA % vigorous PA	X	X
Variables that affect outcome without affecting exposure	Age	X	
	Education	X	
	Annual family income	X	
	Marital status	X	
	Ethnicity	X	
	Smoking status	X	X
	Pain	X	X
	Fatigue		X
	Cancer worry	X	X
Variables that affect both outcome and exposure	Depressive symptoms	X	X
	Stress	X	X

% light, moderate, vigorous intensity PA = proportion of time spent in physical activity of varying intensity (light, moderate, vigorous)

Associations between PAS (T3) and light, moderate, and vigorous intensity PA (T4) were estimated using linear regression models weighted by the inverse of the treatment probability based on propensity scores in each of the 20 imputed data sets. Coefficients from the 20 linear regressions and 95% confidence intervals were pooled according to Rubin’s rules [40]. All analyses were performed with the R statistical analysis software, version 4.0.1 [41]. Multiple imputation was conducted with the MICE package, version 3.13.0 [42]. Propensity score analyses were performed using the MatchThem package [43].

## Results

Of the 199 participants recruited (T1), 19 were lost to follow-up at T4 (9.5%) and removed from the analytical sample. Of the remaining 180 participants (mean age = 55.0 years (SD = 10.1), 85% White) included in the analyses, 125 (69.4%) had missing data on at least one variable. The proportion of participants with missing data varied across variables and ranged from 1.1% to 17.8%. The majority of participants held a post-secondary degree (70.4%) and had a median family income of $68,500 ($9,000-$2,000,000). At enrollment, participants had completed active treatments for an average of 3.5 months (SD = 2.3). Characteristics of the sample appear in Table 2.

**Table 2:** Baseline characteristics of participants from the *Life After Breast Cancer*: *Moving On* study (2010–2018) included in the analysis (n = 180).

Baseline characteristics		Missing n (%)
Age (years), mean (SD)	55.0 (10.1)	
Annual family income (CDN$), median (range)	68,500 (9,000–2,000,000)	32 (17.8)
White, n (%)	153 (85.0)	
Education, n (%)		
<High School diploma	10 (5.6)	
High School diploma	27 (15.0)	
Some post-secondary education	15 (8.3)	
College/technical/certificate	35 (19.4)	
University diploma	50 (27.8)	
Postgraduate diploma	43 (23.9)	
Marital status, n (%)		
Single	27 (15.0)	
Married/Common Law	113 (62.8)	
Separated	4 (2.2)	
Divorced	25 (13.9)	
Widow	11 (6.1)	
Cancer stage, n (%)		
I	76 (42.2)	
II	71 (39.4)	
III	33 (18.3)	
Time since end of treatments (months), mean (SD)	3.5 (2.3)	
Smoking status, n (%)		5 (2.8)
Smokes daily	5 (2.8)	
Smokes occasionally	6 (3.3)	
Does not smoke	164 (91.1)	
% Light PA (% of day), mean (SD)	20.0 (5.3)	2 (1.1)
% Moderate PA (% of day), mean (SD)	1.9 (1.4)	2 (1.1)
% Vigorous PA (% of day), mean (SD)	0.1 (0.3)	2 (1.1)
PAS T3 (1–7), mean (SD)	3.5 (1.7)	13 (7.2)
Depressive symptoms (1–4), mean (SD)	1.7 (0.5)	3 (2.2)
Cancer worry (1–5), mean (SD)	2.6 (0.6)	3 (1.7)
Pain (0–12), mean (SD)	1.9 (1.6)	1 (0.6)
Stress (0–4), mean (SD)	2.6 (0.5)	4 (2.2)
Fatigue (0–10), mean (SD)	3.3 (2.3)	5 (2.8)

PA = Physical Activity computed as a proportion of accelerometer wear time spent in light, moderate and vigorous intensity physical activity (continuous)

Linear regression analyses performed on the imputed samples weighted by the inverse probability of treatment based on propensity scores revealed null associations between PAS and low ($\hat{\beta }$(95%CI) = -0.09 (-0.68, 0.49)), moderate ($\hat{\beta }$(95%CI) = -0.03 (-0.17, 0.11)), and vigorous ($\hat{\beta }$(95%CI) = -0.00 (-0.03, 0.02)) intensity PA (Table 3). In other words, an increase in the PAS score does not translate into a statistically significant increase or decrease in light, moderate, or vigorous PA. Precise confidence intervals suggest either no association between PAS and PA or a very small effect of PAS on PA.

**Table 3:** Results from linear regression models assessing the association between perceived autonomy support and light, moderate, and vigorous intensity PA (n = 180).

	$\hat{\beta }$ (95% CI)
Model 1: Light intensity PA	-0.09 (-0.68, 0.49)
Model 2: Moderate intensity PA	-0.03 (-0.17, 0.11)
Model 3: Vigorous intensity PA	0.00 (-0.03, 0.02)

PA = Physical Activity computed as a proportion of accelerometer wear time spent in (light, moderate, vigorous) intensity physical activity (continuous)

## Discussion

Using linear regression analyses combined with IPTW, we estimated the relationship between PAS from healthcare professionals at 3 months after enrollment (T3) and time spent in light, moderate, and vigorous intensity PA at 9 months after enrollment (T4), hypothesizing that higher PAS would be associated with higher levels of PA. The results of the present study suggest that there is no association between PAS and PA of all intensities in our sample of women recently diagnosed with breast cancer.

PA is known to improve quality of life and physical and psychological health of women treated for breast cancer, but few meet PA recommendations [2–5]. Research stemming from SDT indicates that patient-professional interactions that support autonomy for PA may encourage women who have been treated for breast cancer to adopt and maintain regular PA [13–15]. Our results are consistent with those from both observational and experimental studies suggesting that autonomy support is not associated with PA [44–46]. It is thus possible that the present findings reflect the fact that PAS does not contribute to supporting PA in women who have been recently treated for breast cancer.

SDT literature shows that PAS affects motivation to engage in PA, but it may not affect other barriers to PA experienced by women who have been treated for breast cancer and thus, PAS might not influence PA behaviour [47, 48]. Literature shows that time since diagnosis or end of treatment is not associated with PA trajectories [7, 49, 50]. Time since diagnosis or treatment completion, however, is associated with fatigue symptoms; they are more frequent and intense in the months following treatment completion [49]. For some patients, symptoms remain persistent even 18 months after the end of treatments [49, 50]. Fatigue often occurs alongside other symptoms, such as depressive symptoms or cancer-related concerns, which also act as barriers to PA [49]. Added to these are other barriers, such as lack of knowledge about PA, lack of time to be active and lack of access to tailored and supervised PA programs [9, 49]. The women in our sample were, on average, 15 months post-treatment when we measured the outcome of interest (PA at T4). They were therefore in a phase where several barriers to PA were occurring simultaneously. Thus, participants in our sample may have decreased their PA levels regardless of the autonomy support received because of the greater barriers to PA encountered during this period. Indeed, it would appear that clinicians’ PA support and recommendations may need to be coupled with locally accessible, cancer-specific PA services and resources to increase PA participation in patients [51]. Thus, PAS might improve motivation for PA, but it does not necessarily translate into PA adoption.

### Study strengths and limitations

The present study has several strengths. The study included a sample of women recruited rapidly after the end of active treatment, enabling us to study this pivotal period for the promotion of physical activity. The use of propensity score analysis allowed optimized control of confounding. We used the Covariate Balancing Propensity Score method to estimate propensity scores. Although this method is rather robust to model misspecification, certain covariate selection strategies are to be preferred to reduce the bias associated with variable measurement reliability [33, 52]. One such strategy is to include repeatedly measured variables in the model [33]. In this longitudinal study, we included several variables that were measured at both T1 and T2 (i.e., prior to the exposure measured at T3), thus improving the model’s ability to reduce measurement bias.

This study also has several limitations. Device-measured PA using an accelerometer limits recall bias associated with self-reported measures. However, the predetermined thresholds for the general population that have been used to identify time spent in light, moderate, and vigorous PA are currently being questioned for their use with women with breast cancer [27, 53, 54]. The thresholds for moderate- and vigorous-intensity PA would be lower for this population than for the general population [53, 54]. Thus, the use of the Freedson thresholds may have underestimated time spent in moderate and vigorous intensity PA, thereby reducing the number of participants identified as having engaged in these activities and the time spent in each. This potential measurement error of the outcome would increase the variance of the estimate of interest, but it would not bias it towards zero. The use of a self-reported questionnaire to measure PAS may be subject to misclassification because of participant recall errors. Women who are more active or who have seen their healthcare provider recently or for a longer period might have better recall of the autonomy support they received.

## Conclusions

PAS is not associated with device-measured PA in women who have been treated for breast cancer. Although PAS has been shown to increase motivation for PA, literature also show that PAS does not effectively translate into behaviour change, PA adherence and maintenance. Women who have recently completed treatment for breast cancer face many challenges in engaging in PA and healthcare providers need guidance and resources to support their patients in adopting regular PA and meeting PA recommendations. We expect results to be different in a prehabilitation population, or in a population that has been post-treatment for a longer period and has fewer barriers to exercise. PA programs for people with cancer show promise, but a more comprehensive approach is needed to increase the availability and accessibility of these programs, which are currently limited. In light of the present findings, there is a need to develop and test interventions that aim to act on the many barriers to PA, including those encountered by healthcare professionals and organizations in their implementation [51]. Our findings underscore the importance of considering diverse factors influencing PA in breast cancer survivors and the necessity for multifaceted strategies to promote physical activity in this population.

## Supporting information

S1 Fig Love plot of standardized mean differences between exposed and unexposed participants for each variable in model and for the unweighted and weighted sample. (DOCX)

S1 Table Comparison of characteristics between *Life After Breast Cancer*: *Moving On* (2010–2018) study participants with missing data for at least one variable of interest and participants with complete data for all variables of interest. (DOCX)

S2 Table Baseline characteristics of participants in the *Life After Breast Cancer*: *Moving On* study (2010–2018) in the original dataset and in imputed dataset #3. (DOCX)
