# Supplementary material for: Perceived autonomy support from healthcare professionals and physical activity among breast cancer survivors: A propensity score analysis
Source: PLoS One. 2023 Dec 22;18(12):e0295751. doi: 10.1371/journal.pone.0295751 (PMC10745153; doi:10.1371/journal.pone.0295751)
Supplement: S1 Fig — (DOCX) [file pone.0295751.s001.docx]

**S1 Figure.** Love plot of standardized mean differences between exposed and unexposed participants for each variable in model and for the unweighted and weighted sample.Notes: The dotted lines represent the 0.1 threshold beyond which the variables are not considered balanced.


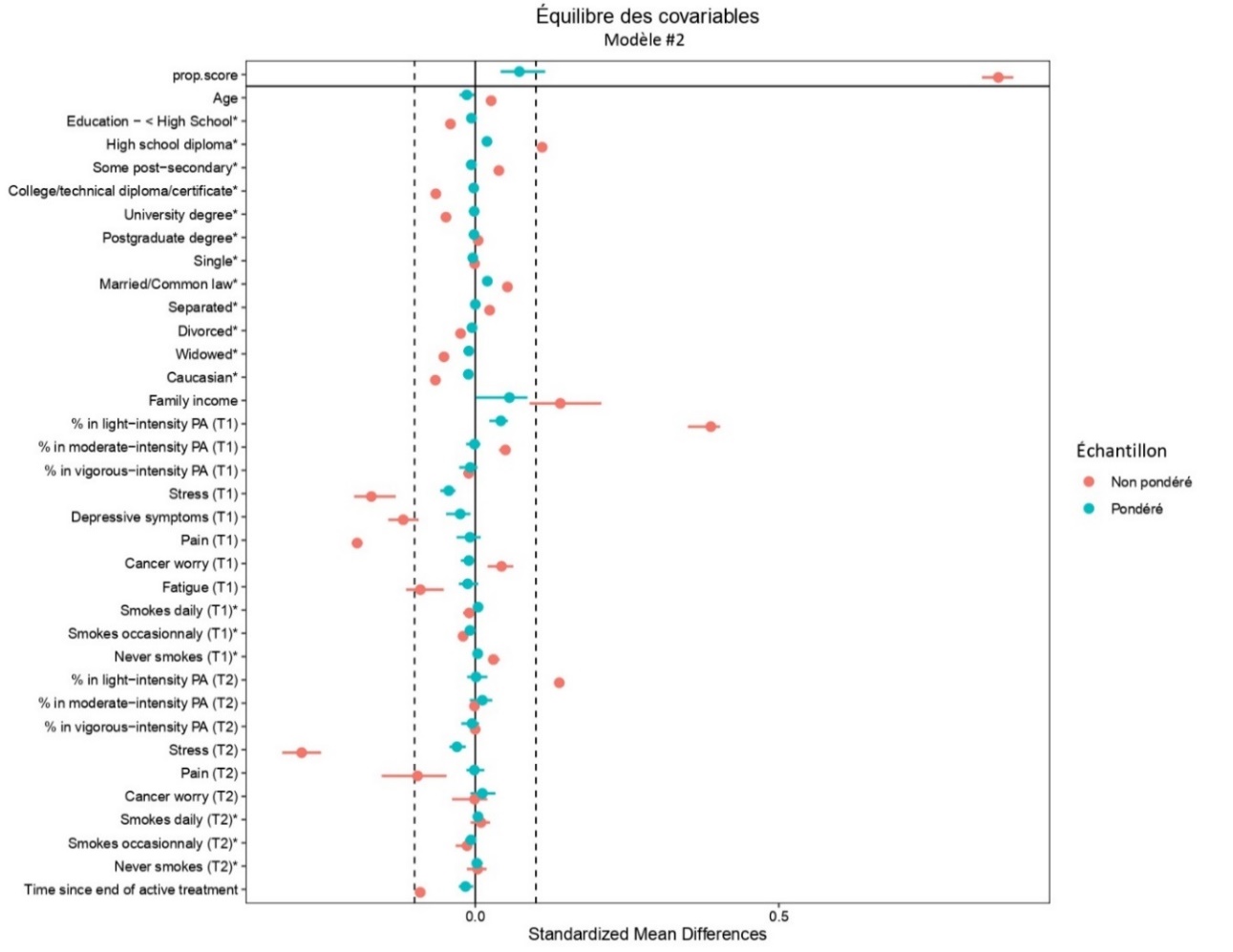


Sample

Unweighted

Weighted with inverse probability of treatment weighting (IPTW)
