# Supplementary material for: Perceived autonomy support from healthcare professionals and physical activity among breast cancer survivors: A propensity score analysis
Source: PLoS One. 2023 Dec 22;18(12):e0295751. doi: 10.1371/journal.pone.0295751 (PMC10745153; doi:10.1371/journal.pone.0295751)
Supplement: S1 Table — (DOCX) [file pone.0295751.s002.docx]

**S1 Table.** Comparison of characteristics between *Life After Breast Cancer: Moving On* (2010-2018) study participants with missing data for at least one variable of interest and participants with complete data for all variables of interest.

|  | Participants with missing data on at least one variable (n=125) | Participants with complete data (n=55) | *p*-value |
| --- | --- | --- | --- |
| Age T1 (years), mean (SD) | 55.89 (11.28) | 52.95 (10.20) | .10 |
| White T1, n (%) | 104 (83.2) | 49 (89.1) | .43 |
| Education T1, n (%) |  |  | .26 |
| High school diploma or less | 25 (20.0) | 12 (21.8) |  |
| College/technical/certificate | 39 (31.2) | 11 (20.0) |  |
| University diploma | 37 (29.6) | 13 (23.6) |  |
| Postgraduate diploma | 24 (19.2) | 19 (34.5) |  |
| Annual family income T1 (CDN$), mean (SD) | 101,471 (206,988) | 110,632 (168,929) | .78 |
| Annual family income T1 (CDN$), median (range) | 70,000 (9,000-2,000,000) | 65,000 (13,000-1,200,000) |  |
| Marital status, n (%) |  |  |  |
| Single | 17 (13.6) | 10 (18.2) | .14 |
| Married/common law | 80 (64.0) | 33 (60.0) |  |
| Separated | 4 (3.2) | 0 (0.0) |  |
| Divorced | 14 (11.2) | 11 (20.0) |  |
| Widow | 10 (8.0) | 1 (1.8) |  |
| BMI T1 (kg/m^2^), mean (SD) | 26.71 (15.88) | 25.27 (5.42) | .12 |
| Smoking status T1, n (%) |  |  | .31 |
| Smokes daily | 5 (4.2) | 0 (0.0) |  |
| Smokes occasionally | 4 (3.3) | 2 (3.6) |  |
| Does not smoke | 111 (92.5) | 53 (96.4) |  |
| Cancer stage T1, n (%) |  |  | **.047** |
| I | 56 (44.8) | 20 (36.4) |  |
| II | 52 (41.6) | 19 (34.5) |  |
| III | 17 (13.6) | 16 (29.1) |  |
| Surgery T1, n (%) | 120 (96.0) | 53 (96.4) | .99 |
| Chemotherapy T1, n (%) | 74 (59.2) | 40 (72.7) | .12 |
| Radiotherapy T1, n (%) | 112 (89.6) | 50 (90.9) | .99 |
| Hormone therapy T1, n (%) | 67 (53.6) | 27 (49.1) | .69 |
| Time since end of treatment T1 (months), mean (SD) | 3.54 (2.33) | 3.27 (2.42) | .49 |
| % light PA T1, mean (SD) | 19.79 (5.08) | 20.54 (5.85) | .35 |
| % moderate PA T1, mean (SD) | 1.88 (1.40) | 1.82 (1.31) | .77 |
| % vigorous PA T1, mean (SD) | 0.08 (0.30) | 0.11 (0.30) | .53 |
| Fear T1 (1-5), mean (SD) | 2.30 (0.95) | 2.30 (0.98) | .99 |
| Stress T1 (0-4), mean (SD) | 2.63 (0.55) | 2.47 (0.54) | .07 |
| Depressive symptoms T1 (1-4), mean (SD) | 1.75 (0.54) | 1.72 (0.49) | .66 |
| Pain T1 (0-12), mean (SD) | 1.92 (1.54) | 1.76 (1.88) | .55 |
| Fatigue T2 (0-10), mean (SD) | 3.38 (2.17) | 3.12 (2.51) | .48 |
| Cancer worry T1 (1-4), mean (SD) | 2.60 (0.81) | 2.56 (0.65) | .73 |
| PAS T3 (1-7), mean (SD) | 3.39 (1.77) | 3.71 (1.55) | .26 |
| Amotivation T3 (0-6), mean (SD) | 0.29 (0.65) | 0.10 (0.30) | **.05** |
| Negative affect T2 (1-5), mean (SD) | 1.80 (0.73) | 1.52 (0.48) | **.01** |
| % (light. moderate. vigorous) PA = proportion of time spent in physical activity of varying intensity (light. moderate. vigorous). SD= Standard deviation. BMI= Body mass index. PAS = Perceived autonomy support.  **Notes**: Unless otherwise indicated, variables are measured at baseline (T1). In **bold**, statistically significant variables at a threshold of 0.05 following t-tests for continuous data and chi-square and Fisher tests for categorical data. | | | |
