# Supplementary material for: Perceived autonomy support from healthcare professionals and physical activity among breast cancer survivors: A propensity score analysis
Source: PLoS One. 2023 Dec 22;18(12):e0295751. doi: 10.1371/journal.pone.0295751 (PMC10745153; doi:10.1371/journal.pone.0295751)
Supplement: S2 Table — (DOCX) [file pone.0295751.s003.docx]

**S2 Table.** Baseline characteristics of participants in the *Life After Breast Cancer: Moving On* study (2010-2018) in the original dataset and in imputed dataset #3.

|  | Original dataset  (n=180) | Imputed dataset #3  (n=180) |  |
| --- | --- | --- | --- |
| Age T1 (years), mean (SD) | 55 (11.02) | 55 (11.02) |  |
| White T1, n (%) | 153 (85.0) | 153 (85.0) |  |
| Education T1, n (%) |  |  |  |
| High school diploma or less | 37 (20.6) | 37 (20.6) |  |
| College/technical/certificate | 50 (27.7) | 50 (27.7) |  |
| University diploma | 50 (27.7) | 50 (27.7) |  |
| Postgraduate diploma | 43 (23.9) | 43 (23.9) |  |
| Annual family income T1 (CDN$), mean (SD)† | 104,876 (193,175) | 99,448 (176,138) |  |
| Annual family income T1 (CDN$), median (range) | 68,500 (9,000-2,000,000) | 66,000 (9,000-2,000,000) |  |
| Marital status T1, n (%) |  |  |  |
| Single | 27 (13.6) | 27 (13.6) |  |
| Married/common law | 128 (64.3) | 128 (64.3) |  |
| Separated | 5 (2.5) | 5 (2.5) |  |
| Divorced | 28 (14.1) | 28 (14.1) |  |
| Widow | 11 (5.5) | 11 (5.5) |  |
| BMI T1 (kg/m^2^), mean (SD) | 26.7 (5.77) | 26.7 (5.77) |  |
| Smoking status T1, n (%)† |  |  |  |
| Smokes daily | 5 (2.8) | 5 (2.8) |  |
| Smokes occasionally | 6 (3.3) | 6 (3.3) |  |
| Does not smoke | 164 (91.1) | 169 (93.9) |  |
| Cancer stage T1, n (%) |  |  |  |
| I | 76 (42.2) | 76 (42.2) |  |
| II | 71 (39.4) | 71 (39.4) |  |
| III | 33 (18.3) | 33 (18.3) |  |
| Surgery T1, n (%) | 173 (96.1) | 173 (96.1) |  |
| Chemotherapy T1, n (%) | 114 (63.3) | 114 (63.3) |  |
| Radiotherapy T1, n (%) | 162 (90.0) | 162 (90.0) |  |
| Hormone therapy T1, n (%) | 94 (52.2) | 94 (52.2) |  |
| Time since end of treatment T1 (months), mean (SD) | 3.46 (2.35) | 3.46 (2.35) |  |
| % light PA T1, mean (SD)† | 20.02 (5.32) | 19.99 (5.30) |  |
| % moderate PA T1, mean (SD)† | 1.86 (1.37) | 1.84 (1.37) |  |
| % vigorous PA T1, mean (SD)† | 0.09 (0.30) | 0.09 (0.30) |  |
| Fear T1 (1-5), mean (SD)† | 2.30 (0.96) | 2.29 (0.96) |  |
| Stress T1 (0-4), mean (SD)† | 2.58 (0.55) | 2.58 (0.55) |  |
| Depressive symptoms T1 (1-4), mean (SD)† | 1.74 (0.52) | 1.74 (0.52) |  |
| Pain T1 (0-12), mean (SD)† | 1.87 (1.65) | 1.86 (1.65) |  |
| Fatigue T2 (0-10), mean (SD)† | 3.30 (2.28) | 3.31 (2.29) |  |
| Cancer worry T1 (1-4), (SD)† | 2.59 (0.76) | 2.59 (0.76) |  |
| % (light. moderate. vigorous) PA = proportion of time spent in physical activity of varying intensity (light. moderate. vigorous). SD= Standard deviation. BMI= Body mass index  † Imputed variables | | | |
